# Using the UPOINT system to manage men with chronic pelvic pain syndrome

**DOI:** 10.1080/2090598X.2021.1955546

**Published:** 2021-07-23

**Authors:** Darren J. Bryk, Daniel A. Shoskes

**Affiliations:** Department of Urology, Glickman Urological and Kidney Institute, Cleveland Clinic Foundation, Cleveland, OH, USA

**Keywords:** Chronic pelvic pain syndrome, chronic prostatitis, UPOINT, prostate, multimodal therapy

## Abstract

**Objective:**

: To outline our approach for the evaluation and management of patients with chronic prostatitis and chronic pelvic pain syndrome (CP/CPPS) based on our interpretation and application of currently available evidence.

**Methods:**

: CP/CPPS in men is a medical condition that plagues both the patient and the practitioner, as it is widely believed to be poorly understood and difficult to treat. While pelvic pain is typically the predominant symptom, many men may exhibit voiding symptoms, sexual dysfunction and psychiatric complaints. Still, most studies of CP/CPPS management have evaluated singular treatments, without focussing on individual patients’ clinical phenotypes. This is a clinically practical mini-review based on the authors’ interpretation and application of currently available evidence related to management of CP/CPPS.

**Results:**

: Patient evaluation should consist of history and physical examination (with focus on the genitourinary and digital rectal examination), laboratory tests (including urine analysis and urine culture with consideration of pre- and post-prostate massage urine cultures), post-void residual, and questionnaires including the National Institutes of Health Chronic Prostatitis Symptoms Index, which helps assess symptom severity and treatment response. Once CP/CPPS is diagnosed, the UPOINT phenotype system, which classifies patients into six domains: Urinary, Psychosocial, Organ Specific, Infectious, Neurological/systemic and Tenderness of skeletal muscles, is used to guide treatment. Each domain is characterised by specific complaints and thus is responsive to distinct treatments. As patients may be grouped into multiple domains, each patient’s overall multimodal treatment can vary.

**Conclusion:**

: Using the UPOINT phenotype system is a holistic approach that can yield significant benefits for patients with CP/CPPS.

## Introduction

Chronic prostatitis and chronic pelvic pain syndrome (CP/CPPS) in men is a medical condition that plagues both the patient and the practitioner, as it is widely believed to be poorly understood and difficult to treat. The prevalence of CP/CPPS or prostatitis-like symptoms has been reported to range from 2% to 10% [1–5]. In order to improve the diagnosis and treatment of prostatitis, the National Institutes of Health (NIH) updated the classification of four prostatitis syndromes based on symptoms rather than aetiology [6]. After acute bacterial prostatitis (Category I) and chronic bacterial prostatitis (Category II), CP/CPPS (Categories IIIA and IIIB) is distinguished by urological pain in the absence of uropathogenic bacteria. There have been other similar definitions of CP/CPPS, including pelvic pain present for at least 3 of the preceding 6 months with no other identifiable cause [7]. Rees et al. [8], in a consensus guideline from the Prostatitis Expert Reference Group, separated CP/CPPS into an early stage, defined as persistent or recurrent symptoms for <6 months and antibiotic naïve; and a late stage, defined as persistent or recurrent symptoms for >6 months and refractory to initial lines of pharmacotherapy. While pain is typically the predominant symptom, many men may exhibit obstructive or irritative voiding symptoms, sexual dysfunction and psychiatric complaints. Still, most studies of CP/CPPS management have evaluated singular treatments, without focussing on individual patients’ clinical phenotypes.

With the understanding that CP/CPPS is a medical syndrome with a wide range of clinical manifestations, the UPOINT phenotype system (Urinary symptoms [U], psychosocial dysfunction [P], organ-specific symptoms [O], infection-related symptoms [I], neurological/systemic conditions [N], tenderness of skeletal muscles [T]), was developed to classify patients with CP/CPPS and create multimodal treatment plans based on their phenotype [9,10]. In this clinically practical mini-review, we outline our approach to the evaluation and management of patients with CP/CPPS, based on our interpretation and application of currently available evidence, with a focus on the UPOINT phenotype classification and multimodal treatment strategies. This should not be interpreted as a rigorous systematic review or meta-analysis. Rather, this article is based on the authors’ interpretation and application of currently available evidence.

## Patient evaluation

As with most medical conditions, the first step in the evaluation of a man with concern about CP/CPPS is a history and physical examination [5]. It should be noted that CP/CPPS is a diagnosis of exclusion, so all other treatable causes of a patient’s pain and LUTS should be evaluated and treated if possible (e.g. ureteric stone, bladder tumour, urethral stricture, etc.) [8,11]. As previously mentioned, pain is the most common complaint. Pain can be present in the perineum, penis, testes, suprapubic area, inguinal region/groin, rectum, abdomen and lower back [8]. Pain may be associated with ejaculation or urination [11]. Patients may also complain about LUTS, including weak stream, straining to void, hesitancy, urgency with or without incontinence and urethral burning; sexual complaints can include erectile dysfunction (ED), haematospermia or other ejaculatory dysfunction and decreased libido [8]. Concomitant psychiatric conditions, such as anxiety and depression, and psychosocial factors, such as pain catastrophising and poor adjustment, are common in this population [8,12], which can impact aetiology but also be the result of chronic pain. Pain complaints may be further exacerbated by pain catastrophising, whereby patients ruminate and magnify their symptoms thus worsening the patient’s quality of life [13].

A focussed physical examination is the next step and should evaluate the penis, scrotum and testes, inguinal regions, perineum, prostate and pelvic floor via a DRE, and any other location for which there is pain [5,8,9,14,15]. The DRE should evaluate for any prostate abnormalities (size, nodule, induration, tenderness) as well as for anorectal abnormalities and for pelvic muscle tenderness or spasm. It should be noted that the finding of a ‘boggy prostate’ is of no diagnostic value [16].

Once the physical examination is complete, the next step is laboratory tests or other diagnostic tools [9,15]. Urine analysis and urine culture should be obtained. During the DRE, prostate massage can be performed to obtain expressed prostate secretions (EPS), which can be cultured, or to obtain a post-massage urine for culture. Pre- and post-massage urine cultures, also known as the ‘two-glass’ test can aid in diagnosis of chronic bacterial prostatitis with similar accuracy as the historical ‘four-glass’ test [17]. As antibiotics can persist in the prostate fluid, cultures should ideally be obtained after being off antibiotics for ≥2 weeks. If appropriate by history, testing for sexually transmitted infections should be included [8]. We routinely measure a post-void residual in all men with pelvic pain or LUTS. The PSA level should be measured as appropriate for age and physical examination. Cystoscopy is indicated if other pathology is suspected (e.g. haematuria, interstitial cystitis) but does not need to be part of the routine evaluation [5]. The key features in men to suggest interstitial cystitis are severe LUTS and pain that worsens with bladder filling and improves with emptying [18].

Patient questionnaires are useful to assess symptom severity and treatment response but are not used to diagnose the condition. The NIH Chronic Prostatitis Symptoms Index (NIH-CPSI) is a validated measure of men with CP and should be administered to all men who are being evaluated for CP/CPPS [19]. It addresses pain, urinary function, and quality of life. This questionnaire is also helpful for use during follow-up visits after treatments have been attempted; a 6-point decline in the NIH-CPSI score has been shown to be an optimal threshold to predict treatment response [20]. Other questionnaires may be used as indicated, including the IPSS (to assess bother of voiding symptoms), Patient Health Questionnaire (to diagnose and assess severity of depression) and Pain Catastrophising Scale (measures negative thoughts associated with pain) [9].

## Multimodal treatment strategy

There have been myriad studies that have evaluated various monotherapies in the treatment of CP/CPPS. Franco et al. [7,21] in two Cochrane reviews of pharmacological and non-pharmacological interventions for the treatment of CP/CPPS, respectively, found low-quality evidence that some monotherapies may provide a small decrease in symptoms, rarely with a decrease in NIH-CPSI score of >6 and the majority with limited long-term outcomes. Even for the helpful therapies, such as α-blockers, large multicentre trials have failed to show benefit, likely because of phenotypic diversity of this syndrome [22].

### The UPOINT phenotype system

**Figure 1.**
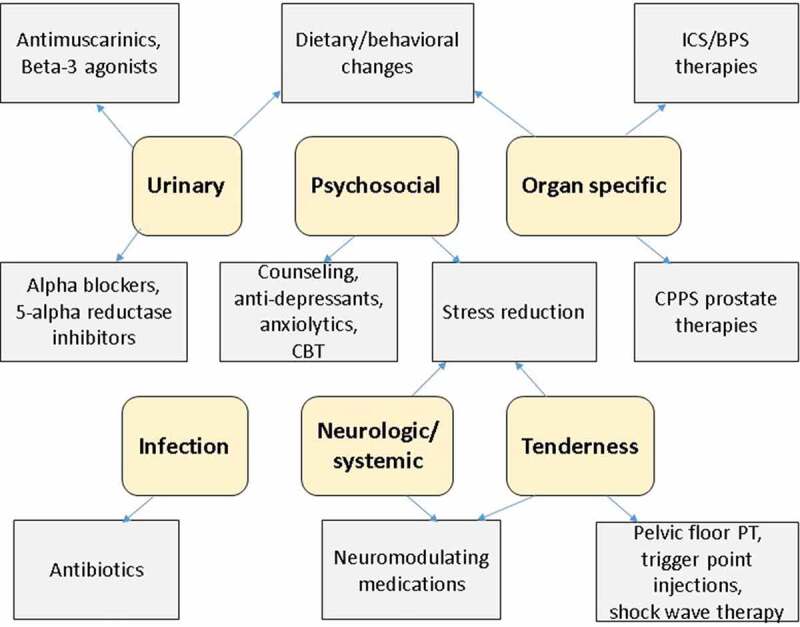
The UPOINT phenotype system treatment guide [11,23]. CBT: cognitive behavioural therapy; IC/BPS: interstitial cystitis/bladder pain syndrome; PT: physical therapy

Once a diagnosis of CP/CPPS has been made, a multimodal approach that addresses a patient’s phenotype (based on his specific complaints) can be formulated [8]. Our approach is the UPOINT system for the clinical phenotyping of chronic pelvic pain, which classifies patients into six domains (Urinary, Psychosocial, Organ Specific, Infectious, Neurological/systemic, and Tenderness of skeletal muscles) based on patient evaluation including patient complaints, physical examination findings, laboratory tests and NIH-CPSI scores and thus guides appropriate therapy [9–11,23]. The number of positive domains in the UPOINT system has been shown to correlate with increasing NIH-CPSI [10,15,24–26]. As seen in Figure 1 [11,23], based on patient phenotypes, the UPOINT system can be used to guide treatment of CP/CPPS. As patients may be grouped into multiple domains, each patient’s overall treatment can vary.

Patients with the ‘**Urinary’** phenotype complain of LUTS, including bothersome nocturia, daytime frequency or urinary urgency, may have an NIH-CPSI urinary score >4 and may have incomplete emptying of the bladder. The ‘Urinary’ domain is often among the most commonly positive domains in men with CPPS, ranging from 60–72% of CPPS populations [3,27,28]. A post-void bladder scan should be obtained in these patients to evaluate for elevated residual urine or urinary retention. Treatments can include behaviour modifications (timed voiding, fluid intake limitation and dietary changes, such as avoiding caffeine) and medications (such as α-blockers, 5α-reductase inhibitors, antimuscarinics and β_3_ agonists) with drug choice based on the predominant urinary complaint [5,8,11,29–31].

Patients in the ‘**Psychosocial’** domain often have depression or depressive symptoms, anxiety, stress and poor coping/adjustment mechanisms; patients may also catastrophise, characterised by a sense of helplessness and hopelessness about the condition and rumination about their symptoms [13]. Patients with CP/CPPS have a high prevalence of psychological issues and may have a history of sexual or other physical abuse, which is associated with poorer quality of life [9,13]. Treatments should include referral to appropriate psychological therapy (including cognitive behavioural therapy), counselling, antidepressants and anxiolytics (prescribed by a mental health specialist), and stress reduction techniques [8,12,32].

The ‘**Organ-specific’** patients have complaints that implicate the prostate and/or bladder as symptom drivers. Prostate-related symptoms can include prostate tenderness to palpation, white blood cells in EPS, haematospermia and prostate calcifications; treatments can include anti-inflammatory phytotherapies such as quercetin, flower pollen and cernilton [5,8,29]. Bladder-related symptoms can include pain with bladder filling that improves with voiding and Hunner lesions seen on cystoscopy. These symptoms suggest a diagnosis of interstitial cystitis/bladder pain syndrome; treatments should follow the algorithm in the AUA guideline on Interstitial Cystitis/Bladder Pain Syndrome [33].

Patients with CP/CPPS are often prescribed empiric antibiotics, which are rarely effective at improving symptoms [9]. The ‘**Infection**’ domain refers to instances where patients have uropathogenic bacteria in urine, EPS or urethra without meeting criteria for UTI or Category I or II prostatitis [6]. *Mycoplasma* and *Ureaplasma* may be such pathogens present that are not commonly tested [23,24]. In the absence of infection, antibiotics will not be helpful [29]; however, for patients in this domain, culture-directed antibiotics are indicated.

The ‘**Neurological/systemic’** patients can be characterised by pain outside the lower abdomen, genitals and pelvis, fibromyalgia, chronic fatigue syndrome, irritable bowel syndrome, and/or other systemic pain complaints [2,9,11]. For these patients, neuromodulators, such as tricyclic antidepressants (amitriptyline), duloxetine, gabapentin and pregabalin, are recommended [8,29]. Cannabinoids can also be used [34]. Chronic opioids should be avoided [35].

Patients in the ‘**Tenderness’** domain have spasm, tenderness and/or trigger points of the pelvis or abdominal muscles diagnosed on DRE and genital and abdominal examinations [9,14]. First-line treatments for these patients are stress reduction and pelvic floor physical therapy [12,36]. Additionally, muscle relaxants, trigger point injections, acupuncture and low-intensity shockwave therapy can be helpful [5,7,37–40].

### Sexual dysfunction and UPOINT ‘S’

Sexual dysfunction, including ED, ejaculatory dysfunction, orgasmic dysfunction and decreased libido, is a common complaint of men with CP/CPPS [8,15,26,41]. As such, Magri et al. [25] in a study assessing the correlation of positive UPOINT domains to NIH-CPSI scores showed that adding a ‘Sexual Dysfunction’ domain, thus creating a UPOINT ‘S’ system, improved the correlation and better characterised the symptom profile in patients with CP/CPPS. Davis et al. [42] assessed 162 men with CP/CPPS and showed that those in the ‘Sexual Dysfunction’ domain had worse quality of life. Further, the number of positive UPOINT domains has been associated with worsened ED symptoms [26,43]. However, other studies do not support the addition of a ‘Sexual Dysfunction’ domain. Samplaski et al. [44] evaluated 100 patients with CP/CPPS; in a multivariate analysis the total number of positive UPOINT domains was strongly associated with NIH-CPSI score but adding a ‘Sexual Dysfunction’ domain did not affect the relationship. More recently, Arda et al. [28] in a retrospective study of 839 patients with CP/CPPS, reaffirmed the positive correlation of UPOINT domains with NIH-CPSI score, but noted no correlation between ED severity and number of positive UPOINT domains or NIH-CPSI score. To date, no studies have prospectively evaluated the use of the UPOINT ‘S’ system in the treatment of CP/CPPS.

### Clinical use of the UPOINT phenotype system

Prior to the development of the UPOINT system, a multimodal approach to the treatment of CP/CPPS was seldom reported. In 2003, Shoskes et al. [45] reported 1-year data on 54 patients with CP/CPPS who were treated with a stepwise multimodal approach; there was a statistically significant decrease in mean NIH-CPSI score from 22.7 to 13.2. Using the UPOINT system to guide treatment of patients’ CP/CPPS has been successful as well (Table 1 [3,24,27,46]). Shoskes et al. [24] prospectively treated 100 patients with CP/CPPS based on the UPOINT system. At a follow-up of ≥6 months, 84% of patients achieved a > 6-point reduction in NIH-CPSI scores with 50% of patients achieving at least a 50% reduction in NIH-CPSI scores. Guan et al. [3] prospectively treated 140 patients with CP/CPPS using the UPOINT system; 75% of patients achieved a ≥ 6-point reduction in NIH-CPSI score. Magri et al. [46] retrospectively reviewed 914 patients with CP/CPPS phenotyped with the UPOINTS system and given a set multimodal therapy; a reduction of ≥6 points in the NIH-CPSI score was noted in 77.5% of the cohort. In a prospective, controlled study, Krakhotkin et al. [27] compared 54 patients treated based on the UPOINT system with 45 patients who received no therapy; after a median 6-month follow-up, the median NIH-CPSI score in the intervention group significantly decreased from 29.8 to 13.9, whereas in the control group there was no significant change in NIH-CPSI score.

**Table 1.** Clinical studies that used the UPOINT phenotype system for CP/CPPS management

Reference	Study type	Sample size, *n*	Follow-up, months	Baseline NIH-CPSI score, mean (SD)	Post-intervention NIH-CPSI score, mean (SD)	*P*	Other notable outcomes
Shoskes et al., 2010 [24]	Prospective cohort	100	≥6	25.2 (6.1)	13.2 (7.2)	<0.001	84% of cohort with ≥6-point decrease in NIH-CPSI score
Guan et al., 2015 [3]	Prospective cohort	140	≥6	22.99 (7.28)	14.29 (5.7)	<0.001	75% of cohort with ≥6-point decrease in NIH-CPSI score
Magri et al., 2015 [46]	Retrospective cohort	914	6, 12 and 18	20.91 (7.12)	6 months: 9.87 (5.71)	<0.001	77.5% with ≥6-point decrease in NIH-CPSI score
					12 months: 8.15 (4.52)		
					18 months: 7.62 (4.13)		
Krakhotkin et al., 2019 [27]	Prospective controlled	99 (54 intervention, 45 control)	Median 6.21	Intervention group: 29.8 (6.1)	13.9 (2.8)	0.025	Mean number positive UPOINT domains decreased from 3.9 to 1.75 (*P* = 0.022) in the intervention group; no change in the control group
				Control group: 29.8 (6.1)	29.8 (5.8)	0.18	

## Conclusion

CP/CPPS is a heterogeneous syndrome with each patient manifesting a variety of symptoms with different aetiologies and progression trajectories [26]. The UPOINT system is a validated mode of treatment that takes into account each individual patient’s phenotypes of disease and guides treatments, often from multiple medical disciplines. The UPOINT system is easy to reproduce and has flexibility to incorporate new data or patient findings. Using the UPOINT phenotype system is a holistic approach that can yield significant benefits for patients with CP/CPPS.

## References

[1] Chen J, Zhang H, Niu D, et al. The risk factors related to the severity of pain in patients with chronic prostatitis/chronic pelvic pain Syndrome. BMC Urol. 2020;20. DOI:10.1186/s12894-020-00729-9. PMC7542966 33028277

[2] Clemens JQ, Mullins C, Ackerman AL, et al. Urologic chronic pelvic pain syndrome: insights from the MAPP Research Network. Nat Rev Urol. 2019;16(3):187–200. 30560936 10.1038/s41585-018-0135-5 PMC6800057

[3] Guan X, Zhao C, Ou ZY, et al. Use of the UPOINT phenotype system in treating Chinese patients with chronic prostatitis/chronic pelvic pain syndrome: a prospective study. Asian J Androl. 2015;17(1):120–123. 25248659 10.4103/1008-682X.138189 PMC4291855

[4] Krieger JN, Lee SWH, Jeon J, et al. Epidemiology of prostatitis. Int J Antimicrob Agents. 2008;31:85–90. 10.1016/j.ijantimicag.2007.08.028 PMC2292121 18164907

[5] Doiron RC, Shoskes DA, Nickel JC. Male CP/CPPS: where do we stand? World J Urol. 2019;37(6):1015–1022. 10.1007/s00345-019-02718-6 30864007

[6] Krieger JN, Nyberg L, Nickel JC. NIH consensus definition and classification of prostatitis [5]. J Am Med Assoc. 1999;282(3):236–237. 10.1001/jama.282.3.236 10422990

[7] Franco JV, Turk T, Jung JH, et al. Non-pharmacological interventions for treating chronic prostatitis/chronic pelvic pain syndrome. Cochrane Database Syst Rev. 2018;5. DOI:10.1002/14651858.cd012551.pub3. PMC6494451 29757454

[8] Rees J, Abrahams M, Doble A, et al. Diagnosis and treatment of chronic bacterial prostatitis and chronic prostatitis/chronic pelvic pain syndrome: a consensus guideline. BJU International. 2015;116(4):509–525. 25711488 10.1111/bju.13101 PMC5008168

[9] Shoskes DA, Nickel JC, Rackley RR, et al. Clinical phenotyping in chronic prostatitis/chronic pelvic pain syndrome and interstitial cystitis: a management strategy for urologic chronic pelvic pain syndromes. Prostate Cancer Prostatic Dis. 2009;12(2):177–183. 10.1038/pcan.2008.42 18645581

[10] Shoskes DA, Nickel JC, Dolinga R, et al. Clinical Phenotyping of Patients With Chronic Prostatitis/Chronic Pelvic Pain Syndrome and Correlation With Symptom Severity. Urology. 2009;73(3):538–542. 19118880 10.1016/j.urology.2008.09.074

[11] DeWitt-Foy ME, Nickel JC, Shoskes DA. Management of Chronic Prostatitis/Chronic Pelvic Pain Syndrome(Figure presented.). Eur Urol Focus. 2019;5(1):2–4. 10.1016/j.euf.2018.08.027 30206001

[12] Anderson RU, Wise D, Nathanson BH. Chronic Prostatitis and/or Chronic Pelvic Pain as a Psychoneuromuscular Disorder—A Meta-analysis. Urology. 2018;120:23–29. 30056195 10.1016/j.urology.2018.07.022

[13] Huang X, Qin Z, Cui H, et al. Psychological factors and pain catastrophizing in men with chronic prostatitis/chronic pelvic pain syndrome (CP/CPPS): a meta-analysis. Transl Androl Urol. 2020;9(2):485–493. 32420154 10.21037/tau.2020.01.25 PMC7214995

[14] Yang CC, Miller JL, Omidpanah A, et al. Physical Examination for Men and Women With Urologic Chronic Pelvic Pain Syndrome: a MAPP (Multidisciplinary Approach to the Study of Chronic Pelvic Pain) Network Study. Urology. 2018;116:23–29. 10.1016/j.urology.2018.03.021 PMC6237096 29604315

[15] Magistro G, Wagenlehner FME, Grabe M, et al. Contemporary management of chronic prostatitis/chronic pelvic pain Syndrome. Eur Urol. 2016;69(2):286–297. 26411805 10.1016/j.eururo.2015.08.061

[16] Shoskes DA, Berger R, Elmi A, et al. Muscle tenderness in men with chronic prostatitis/chronic pelvic pain syndrome: the chronic prostatitis cohort study. J Urol. 2008;179(2):556–560. 10.1016/j.juro.2007.09.088 PMC2664648 18082223

[17] Nickel JC, Shoskes D, Wang Y, et al. How does the pre-massage and post-massage 2-glass test compare to the meares-stamey 4-glass test in men with chronic prostatitis/chronic pelvic pain Syndrome? J Urol. 2006;176(1):119–124. 10.1016/S0022-5347(06)00498-8 16753385

[18] Arora HC, Shoskes DA. The enigma of men with interstitial cystitis/bladder pain syndrome. Transl Androl Urol. 2015. DOI:10.3978/j.2223-4683.2015.10.01. PMC4708534 26813678

[19] Litwin MS, McNaughton-Collins M, Fowler FJ, et al. The National Institutes of Health chronic prostatitis symptom index: development and validation of a new outcome measure. J Urol. 1999;162(2):369–375. 10411041 10.1016/s0022-5347(05)68562-x

[20] Propert KJ, Litwin MS, Wang Y, et al. Responsiveness of the National Institutes of Health Chronic Prostatitis Symptom Index (NIH-CPSI). Qual Life Res. 2006;15(2):299–305. 10.1007/s11136-005-1317-1 16468084

[21] Franco JVA, Turk T, Jung JH, et al. Pharmacological interventions for treating chronic prostatitis/chronic pelvic pain syndrome. Cochrane Database Syst Rev. 2019;2019. DOI:10.1002/14651858.CD012552.pub2. PMC6778620 31587256

[22] Nickel JC, Krieger JN, McNaughton-Collins M, et al. Alfuzosin and Symptoms of Chronic Prostatitis–Chronic Pelvic Pain Syndrome. N Engl J Med. 2008;359(25):2663–2673. 10.1056/NEJMoa0803240 PMC2815340 19092152

[23] Shoskes D. UPOINT System for the Clinical Phenotyping of Chronic Pelvic Pain. (Chronic Prostatitis/Chronic Pelvic Pain Syndrome, Interstitial Cystitis, Painfull Bladder Syndrome) 2009. http://www.upointmd.com/ (2021 Jan 11).

[24] Shoskes DA, Nickel JC, Kattan MW. Phenotypically Directed Multimodal Therapy for Chronic Prostatitis/Chronic Pelvic Pain Syndrome: a Prospective Study Using UPOINT. Urology. 2010;75(6):1249–1253. 20363491 10.1016/j.urology.2010.01.021

[25] Magri V, Wagenlehner F, Perletti G, et al. Use of the UPOINT chronic prostatitis/chronic pelvic pain syndrome classification in European patient cohorts: sexual function domain improves correlations. J Urol. 2010;184(6):2339–2345. 10.1016/j.juro.2010.08.025 20952019

[26] Zhao Z, Zhang J, He J, et al. Clinical Utility of the UPOINT Phenotype System in Chinese Males with Chronic Prostatitis/Chronic Pelvic Pain Syndrome (CP/CPPS): a Prospective Study. PLoS One. 2013. DOI:10.1371/journal.pone.0052044. PMC3547952 23349680

[27] Krakhotkin DV, Chernylovskyi VA, Bakurov EE, et al. Evaluation of influence of the UPOINT-guided multimodal therapy in men with chronic prostatitis/chronic pelvic pain syndrome on dynamic values NIH-CPSI: a prospective, controlled, comparative study. Ther Adv Urol. 2019;11:175628721985727. 10.1177/1756287219857271 PMC6595637 31263510

[28] Arda E, Cakiroglu B, Tas T, et al. Use of the UPOINT Classification in Turkish Chronic Prostatitis or Chronic Pelvic Pain Syndrome Patients. Urology. 2016;97:227–231. 10.1016/j.urology.2016.07.023 27476153

[29] Polackwich AS, Shoskes DA. Chronic prostatitis/chronic pelvic pain syndrome: a review of evaluation and therapy. Prostate Cancer Prostatic Dis. 2016;19(2):132–138. 26951713 10.1038/pcan.2016.8

[30] Sung YH, Jung JH, Ryang SH, et al. Clinical significance of national institutes of health classification in patients with chronic prostatitis/chronic pelvic pain syndrome. Korean J Urol. 2014;55(4):276–280. 24741418 10.4111/kju.2014.55.4.276 PMC3988440

[31] Gallo L. Effectiveness of diet, sexual habits and lifestyle modifications on treatment of chronic pelvic pain syndrome. Prostate Cancer Prostatic Dis. 2014;17(3):238–245. 10.1038/pcan.2014.18 24819236

[32] Wang J, Liang K, Sun H, et al. Psychotherapy combined with drug therapy in patients with category III chronic prostatitis/chronic pelvic pain syndrome: a randomized controlled trial. Int J Urol. 2018;25(8):710–715. 10.1111/iju.13706 29862568

[33] Hanno PM, Erickson D, Moldwin R, et al. Diagnosis and treatment of interstitial cystitis/bladder pain syndrome: AUA guideline amendment. J Urol. 2015;193(5):1545–1553. 10.1016/j.juro.2015.01.086 25623737

[34] Tripp DA, Curtis Nickel J, Katz L, et al. A survey of cannabis (marijuana) use and self-reported benefit in men with chronic prostatitis/chronic pelvic pain syndrome. J Can Urol Assoc. 2014;8(11–12):901. 10.5489/cuaj.2268 PMC4277530 25553163

[35] Bharucha AE, Lee TH. Anorectal and Pelvic Pain. Mayo Clin Proc. 2016;91(10):1471–1486. 10.1016/j.mayocp.2016.08.011 PMC5123821 27712641

[36] Polackwich AS, Li J, Shoskes DA. Patients with pelvic floor muscle spasm have a superior response to pelvic floor physical therapy at specialized centers. J Urol. 2015;194(4):1002–1006. 10.1016/j.juro.2015.03.130 25912491

[37] Tadros NN, Shah AB, Shoskes DA. Utility of trigger point injection as an adjunct to physical therapy in men with chronic prostatitis/chronic pelvic pain syndrome. Transl Androl Urol. 2017;6(3):534–537. 28725596 10.21037/tau.2017.05.36 PMC5503970

[38] Li G, Man L. Low-intensity extracorporeal shock wave therapy for III B chronic pelvic pain syndrome. Transl Androl Urol. 2020;9(3):1323–1328. 32676416 10.21037/tau.2020.04.07 PMC7354340

[39] Qin Z, Zang Z, Zhou K, et al. Acupuncture for Chronic Prostatitis/Chronic Pelvic Pain Syndrome: a Randomized, Sham Acupuncture Controlled Trial. J Urol. 2018;200(4):815–822. 10.1016/j.juro.2018.05.001 29733836

[40] Pajovic B, Radojevic N, Dimitrovski A, et al. Comparison of the efficiency of combined extracorporeal shock-wave therapy and triple therapy versus triple therapy itself in Category III B chronic pelvic pain syndrome (CPPS). Aging Male. 2016;19(3):202–207. 10.1080/13685538.2016.1197899 27380504

[41] Li HJ, Kang DY. Prevalence of sexual dysfunction in men with chronic prostatitis/chronic pelvic pain syndrome: a meta-analysis. World J Urol. 2016. DOI:10.1007/s00345-015-1720-3. PMC4921105 26546073

[42] Davis SNP, Binik YM, Amsel R, et al. Is a sexual dysfunction domain important for quality of life in men with urological chronic pelvic pain syndrome? Signs “uPOINT” to yes. J Urol. 2013;189(1):146–151. 10.1016/j.juro.2012.08.083 23164384

[43] Magri V, Perletti G, Montanari E, et al. Chronic prostatitis and erectile dysfunction: results from a cross-sectional study. Arch Ital Di Urol e Androl. 2008; 80(4):172-5 19235435

[44] Samplaski MK, Li J, Shoskes DA. Inclusion of erectile domain to UPOINT phenotype does not improve correlation with symptom severity in men with chronic prostatitis/chronic pelvic pain syndrome. Urology. 2011. DOI:10.1016/j.urology.2011.04.016. 21664651

[45] Shoskes DA, Hakim L, Ghoniem G, et al. Long-term results of multimodal therapy for chronic prostatitis/chronic pelvic pain syndrome. J Urol. 2003;169(4):1406–1410. 10.1097/01.ju.0000055549.95490.3c 12629373

[46] Magri V, Marras E, Restelli A, et al. Multimodal therapy for category III chronic prostatitis/chronic pelvic pain syndrome in UPOINTS phenotyped patients. Exp Ther Med. 2015;9(3):658–666. 10.3892/etm.2014.2152 PMC4316954 25667610

